# Evaluation of Cd^2+^ stress tolerance in transgenic rice overexpressing *PgGPx* gene that maintains cellular ion and reactive oxygen species homeostasis

**DOI:** 10.1371/journal.pone.0273974

**Published:** 2022-09-06

**Authors:** Tahmina Islam, M. K. Reddy

**Affiliations:** 1 Crop Improvement Group, International Centre for Genetic Engineering and Biotechnology, New Delhi, India; 2 Plant Breeding and Biotechnology Laboratory, Department of Botany, University of Dhaka, Dhaka, Bangladesh; University of Innsbruck, AUSTRIA

## Abstract

Non-essential toxic heavy metal like cadmium (Cd^2+^) interferes with the plant growth and development in many ways. Cd^2+^ travels via plant transportation system, specifically through xylem and may integrate into the food chain causing unfavorable condition in human health. Therefore, strategies to develop Cd^2+^ tolerance and less accumulation in the plant system require urgent attention. Peroxidase gene family is known for metal ions transportation including Cd^2+^ and thus plays an important role in ion homeostasis. Previously, we have reported the presence of a Cd^2+^ dependent functional peroxiredoxin from *Pennisetum glaucum* (PgGPx). The present study elucidates the role of this *PgGPx* against Cd^2+^ stress in rice. The transcript levels of *PgGPx* were found to be highly upregulated in response to exogenous Cd^2+^. Moreover, recombinant PgGPx protein showed significant glutathione S-transferase activity *in vitro*. Ectopically expressed *PgGPx* in transgenic rice plants showed tolerance towards Cd^2+^ stress as demonstrated by several physiological indices including shoot and root length, biomass, chlorophyll, and hydrogen peroxide content. Moreover, these transgenic plants also showed enhanced capability to cope up with oxidative stress by enhancing the activity of different antioxidant enzymes including Superoxide dismutase, Catalase, Ascorbate peroxidase, Glutathione peroxidase, Glutathione reductase) in response to Cd^2+^. Hence, maintenance of cellular ion homeostasis and modulation of reactive oxygen species-scavenging pathway are found to be improved by overexpression of *PgGPx* under Cd^2+^ stress. These results will pave the way to develop strategies for engineering Cd^2+^ stress tolerance in economically important crop plants.

## Introduction

Heavy metals are highly reactive and thus toxic to living cells. Due to the industrialization, heavy metals pollution of biosphere has increased by several folds. Accumulation of heavy metals in both soil and plants is affecting agricultural productivity. Based on their functional role in plant growth, heavy metals can be classified into two categories i.e. essential and non-essential. Mineral or metals are required in trace amount for the adequate growth and development, but toxic as soon as the concentration exceeds the threshold. The non-essential metals are toxic to plant development, and these include cadmium (Cd^2+^) and lead. In particular, Cd^2+^ is absorbed from the soil by roots and transported to the shoot, thereby negatively affecting nutrient uptake and ion homeostasis, even at low concentrations [1]. Cd^2+^ toxicity leads to leaf rolling, chlorosis, and imbalanced water uptake in plants. Moreover, it adversely affects various biochemical and physiological processes that include change in the transcriptome and proteome of plants, inhibition of seed germination, shoot and root growth [2, 3], stomatal conductance, transpiration, and rate of photosynthesis [4–6] that ultimately lead to the reduction of yield [7, 8]. In several plant species, Cd^2+^ decreases carbon assimilation by inhibiting the activity of CO_2_ fixing photosynthetic enzymes [9]. Moreover, Cd^2+^- induced the generation of reactive oxygen species (ROS) such as superoxide radicals (O_2_^.-^) and hydrogen peroxide (H_2_O_2_) in isolated plasma membranes, mitochondria and intact root cells [10]. These situations therefore instigate cellular damage in various ways including DNA mutation, protein side chains modification, and destruction of phospholipids [11].

In last few decades, use of phosphate fertilizers, sludge, and irrigation of Cd^2+^ containing water tremendously increased the Cd^2+^ content in arable soil. Furthermore, edible parts of plants such as seeds with enhanced accumulation of Cd^2+^ places humans at a great risk [1]. Hence, necessity of studying plant response mechanisms towards Cd^2+^ stress is meant to be very important. Plants are well equipped with several regulatory mechanisms to control the uptake, accumulation, trafficking, and detoxification of heavy metals [2, 12]. Different signaling pathways including hormones [13], ROS [2] and the mitogen-activated protein kinase (MAPK) phosphorylation cascade [14, 15] have been reported to get activated when exposed to Cd^2+^ stress.

There are several gene families namely vacuolar Cd^2+^/proton antiporter (CAX2 and CAX4), ABC transporter and Cation Diffusion Facilitator (CDF) that are directly involved in the transportation of Cd^2+^ and other heavy metals from the cytoplasm to the vacuole suggesting their importance in Cd^2+^ sequestration. Several genes responded towards oxidative stress and associated with defense mechanisms, have also been studied under Cd^2+^ stress in plants. For instance, glutathione S-transferase (GST), peroxidase (Prx), thioredoxins (Trx), peroxiredoxin (PrxR) and catalase (CAT) confer Cd^2+^ tolerance in plants [16–18]. In addition, cysteine-rich metallothioneins (MT) and phytochelatins (PC) are known as chelating agents which induced under Cd^2+^ exposure. These molecules are basically involved to Cd^2+^ sequestration by binding Cd^2+^ ions through S-containing amino acid ligands [7, 19, 20]. However, the exact mechanism how these multigenic families are respond to Cd^2+^ has yet to be investigated. Recent advancement of the molecular mechanism of Cd^2+^ signalling pathways has paved the way to solve the puzzle of the complex Cd^2+^ absorption and sequestration system in plants.

In eukaryotes, the detoxification of Cd^2+^ ions and its tolerance are mainly associated with the activity of several signalling cascades including ABA, DREB, NAC, bZIP along with several antioxidant enzymes including GST, Prx, Trx, PrxR and Cat [1, 17]. Thiols such as glutathione plays a pivotal role in ROS scavenging via GSH-ascorbate cycle and donates the electron to glutathione peroxidase (GPx). Reduced sulphur is stored and remotely transported in this form, that are involved in the detoxification of heavy metals, xenobiotics, and cell cycle regulation [21]. Protein thiols are considered as a protective and important component of regulatory mechanisms for instance thioredoxins. GSTs are well known members of this family that quench reactive molecules. They catalyze the conjugation of GSH to an array of hydrophobic and electrophilic substrates, and thus protecting the cell from oxidative burst. GSTs have been implicated in several cellular processes including tolerance against abiotic stresses [22], heavy metal stress [23], and ultra-violet (UV) radiations [24]. Moreover, it has been reported that GST and peroxidases (Prxs) were among the 20 strongly up-regulated genes with the greatest relative expression in response to Cd^2+^ toxicity [1]. Previously, one of the glutathione peroxidases from *Pennisetum glaucum* (PgGPx) was reported as a Cd^2+^-dependent functional 2-cys peroxidase and overexpression of *PgGPx* in transgenic rice plants provided tolerance against salinity and drought [25]. In the present study, we identified GST activity in PgGPx as a dual function. This dual action of PgGPx propelled us to investigate the functional role of *PgGPx* in response to exogenous Cd^2+^ stress.

## Materials and methods

### Plant growth conditions, and stress treatment

Surface sterilized seeds of pearl millet (*Pennisetum glaucum* (L.) R. Br.) were grown under greenhouse conditions (14/10 h light/dark cycle illumination at 370 μEm^-2^s^-1^ and 30± 2 °C). Fourteen days old seedlings were treated with 100 μM CdCl_2_ for 72 hours according to the previous literature [26]. Shoot samples were collected at eight different time points (0 h, 1 h, 3 h, 6 h, 12 h, 24 h, 48 h, and 72 h) for expression analysis.

Both wild type (*Oryza sativa* L ssp. Japonica) and *PgGPx* overexpressing transgenic rice seeds (Homogenous T_2_ lines) were surface sterilized for 20 min with 1% Bavistin solution and allowed to germinate in a hydroponics system supplemented with Yoshida medium [25]. Fourteen days old rice seedlings were irrigated with or without 100 μM CdCl_2_ for 24 h. To study the effect of Cd^2+^ toxicity on yield, 2-month-old healthy WT and transgenic plants were irrigated with normal water (as control) or 100 μM CdCl_2_ (as stress) and grown until maturity.

### RNA isolation and qRT-PCR

Total RNA was isolated from the stored plant sample and the 1^st^ strand cDNA was prepared according to the manufacturer’s instructions (Thermo Scientific, USA). qRT-PCR was performed using *PgGPx* and housekeeping *Tubulin* gene specific primers (S1 Table). Dissociation curve analysis was performed to specify the amplification with the default parameters. Three technical replicates were used for each sample. The relative expression levels of *PgGPx* was calculated using 2^-ΔΔCT^ method (S1 File) as described previously [25].

### Purification of recombinant PgGPx protein and enzyme kinetics

Recombinant PgGPx protein was purified according to the previous report [25]. Following protein purification, Bradford method [27] was to quantify the protein. The specific glutathione S-transferase (GST, EC 2.5.1.18) activity of PgGPx was measured using reduced glutathione (GSH) and 1-chloro-2,4-dinitrobenzene (CDNB) as substrates by taking absorbance at 340 nm after one minute interval for 5 min. The enzyme activity was expressed as nmol/min/mg protein [28]. Initial velocity of PgGPx was determined using a constant concentration of CDNB (1 mM) and a varying concentration of GSH (0.5 mM to 1.5 mM). Various kinetic parameters of PgGPx were calculated by extrapolating a Lineweaver-Burk plot. The experiment was repeated three times and the data was represented as the average value ± standard deviation (n = 3).

### Physiological and biochemical parameters of *PgGPX* overexpressed transgenic rice plants

Fourteen days old rice seedlings were treated with 100 μM CdCl_2_ for 24 h. Various physiological parameters such as shoot length, root length, and fresh weight were measured from both WT and transgenic plants under control and stressed condition. Relative water content (RWC) [29] and electrolytic leakage [30] were measured from the leaf of WT and transgenic plants as described previously.

The chlorophyll content was measured spectrophotometrically from the leaves after extraction in 80% acetone according the previous report [31]. Leaf tissue (100 mg) was homogenized thoroughly in 1 ml of 80% acetone and centrifuged at 3000 rpm for 2–3 min. The supernatant was retained, and absorbance was recorded using a spectrophotometer at 663 nm and 647 nm. Anthocyanin content was measured according to the previous report [32]. Seedlings were homogenized in Propanol: HCl: water (18:1:81). Samples were boiled in a water bath for 15 mins followed by centrifugation at 10,000 rpm. The absorbance of the supernatant was measured at 535 nm and 650 nm. All measurements were repeated three times (n = 3).

### Visualization of H_2_O_2_ and O_2_^.-^ accumulation

The presence of intercellular H_2_O_2_ was visualized by staining with DAB (3, 3’-diaminobenzidine) solution (1 mg/ml). Plant tissue samples were dipped in appropriate amount of DAB solution and gentle vacuum was applied for 5 min. The plates were covered with aluminium foil and kept in dark for 48 h with gentle shaking of 80–100 rpm. Tissues were treated with series of 90% and 70% ethanol to bleach out the chlorophyll and visualized on a plain white background.

Cellular generation of O_2_^.-^ was visualized histochemically [33]. For histochemical visualization of O_2_^.-^, fresh leaves were dipped at 50 mM Tris-HCl buffer pH 6.4 containing 0.1% Nitroblue tetrazolium (NBT) and 0.1% NADH, for 10–15 min. The leaves were subjected to illumination under high white light to develop the characteristic blue monoformazan precipitation. Stained leaves were photographed against a white fluorescent light background.

### Quantification of total H_2_O_2_

The level of total H_2_O_2_ was quantified according to Velikova et al. [34]. Fresh leaf tissues were extracted with 5 ml trichloroacetic acid (0.1% w/v) in ice cold bath, and the homogenate was centrifuged at 13,000 g for 15 min. Equal volume of sodium phosphate buffer (pH 7.5) and double volume of potassium iodide were added to 0.5 ml supernatant. The absorbance of the sample was measured at 390 nm. H_2_O_2_ content was determined using extinction coefficient (ε = 0.28 μM^-1^ cm^-1^) and expressed as nM of H_2_O_2_ g^-1^ fresh weight of plant tissue. All the measurements were repeated three times (n = 3).

### Total protein extraction and measurement of antioxidant enzymes

Total plant protein was extracted from the collected shoots of both WT and transgenic plants grown under control or Cd^2+^ exposure using an extraction buffer containing 100 mM potassium phosphate buffer, pH 7.0, 50% glycerol, 16 mM MgSO_4_ and 1 mM PMSF [35] at 4°C and quantified by Bradford method [27]. Activity of the antioxidant enzymes such as superoxide dismutase (SOD), ascorbate peroxidase (APX), catalase (CAT), glutathione S-transferase (GST), peroxidases and glutathione reductase (GR) was determined as described previously [36]. Activity of SOD was assayed based on its ability to compete with nitroblue tetrazolium (NBT) for superoxide anions generated by the xanthine-xanthine oxidase. Total protein was taken into 800 μl of phoshate buffer, 50 μl NBT, 0.1 unit of catalase and 0.1 unit of xanthine oxidase. The change in absorbance was followed upto 2 min at 560 nm. APX activity was measured in an assay buffer containing 50 mM phosphate buffer (pH 7.0), 0.5 mM Ascorbate, 0.2mM EDTA and protein extract in a total volume of 1 ml. The rate of ascorbic acid oxidation was initiated by adding 0.5mM H_2_O_2_ and the decrease in absorbance was monitored at 290 nm. Activity of GR was measured in 100 mM potassium phosphate buffer (pH 7.6) containing 1 mM EDTA, 5 mM NADPH, 6 mM 5,5’-dithio-bis (2-nitrobenzoic acid) and 0.2 mM oxidized glutathione (GSSG). The change in absorbance was followed at 412 nm upto 2 min. CAT activity was measured at 50 mM phosphate buffer (pH 7.0) containing 33.5 mM H_2_O_2_ and protein extract. The decrease in absorbance of H_2_O_2_was recorded for 2 min at 240 nm. Activity of GST was measured spectrophotometrically at 344 nm using reduced glutathione and 1-chloro-2,4-dinitrobenzene (CDNB) substrates [28]. All the enzyme activities were repeated three times (n = 3) and expressed as mean ± standard deviation.

### Measurement of photosynthetic parameters

Important parameters for photosynthesis i.e. photosynthesis rate, stomatal conductance and chlorophyll fluorescence (Fv/Fm) were determined from the third to fifth expanded leaves using an infra-red gas analyzer (Li-COR 6400–40, Lincoln, USA) with default settings [25]. All these measurements were repeated three times (n = 3).

### Statistical analysis

Statistical significance was determined using the Analysis of variance (ANOVA) test at P = 0.05 with Bonferroni corrected post hoc analysis.

## Results

### Time dependent transcript profiling of *PgGPx* in response to exogenous Cd^2+^ stress

**Fig 1:**
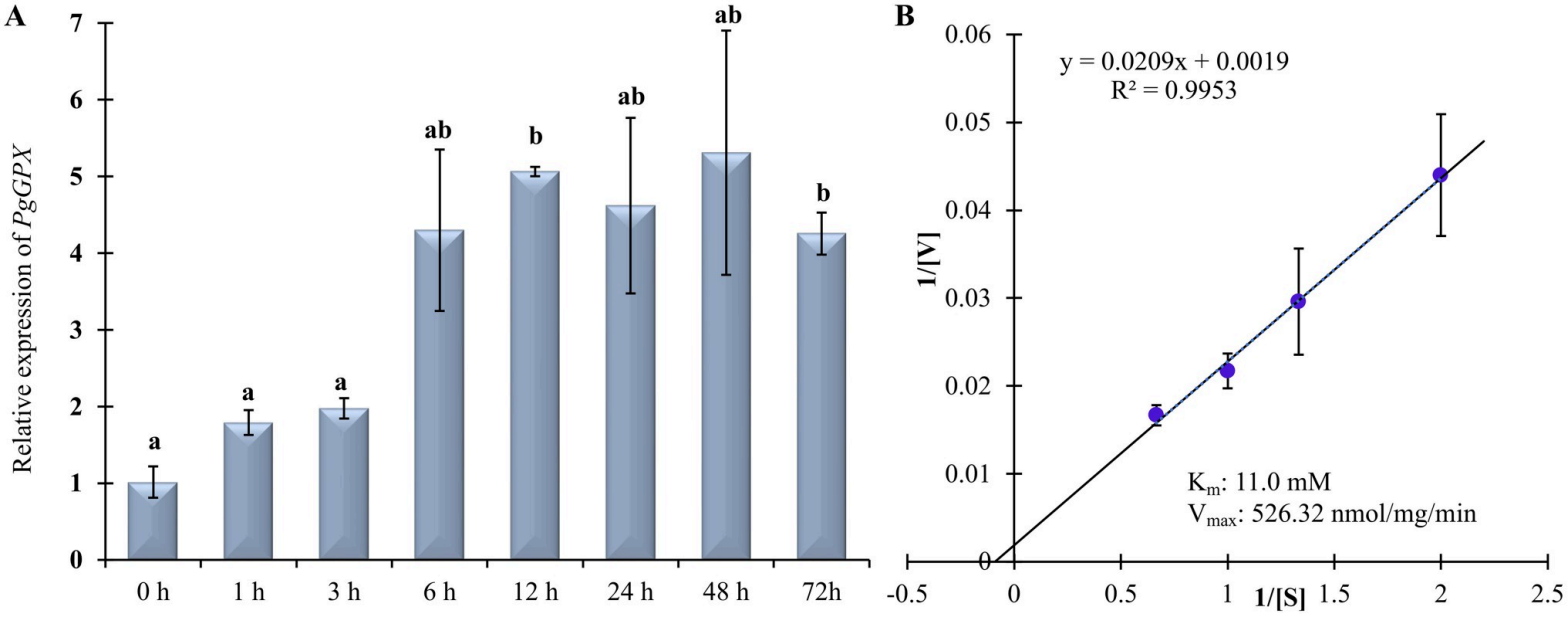
Transcript profiling and enzyme kinetics of PgGPx. (A) A time-dependent expression analysis of *PgGPx* gene was carried out in response to 100 μM CdCl_2_ in *Pennisetum gluccum* seedlings for 72h using quantitative real-time PCR. Relative level of *PgGPx* was normalized with respect to the house keeping *tubulin* gene. Data were presented as mean expression value ± standard deviation of three replicates (n = 3). Different letters above the bar showed significant difference between the treatments and plant genotypes (P < 0.05, ANOVA; post hoc test). (B) PgGPx illustrates GST activity. Hanes-Woolf plot was generated to depict the enzyme kinetics of PgGPx in the presence of varying concentration of reduced glutathione and a constant 1-Chloro-2,4-dinitrobenzene (CNDB). Experiments were repeated three times and the enzyme kinetics values were shown in the inset.

To elucidate the role of *PgGPx* (S1 Text) in response to Cd^2+^ stresses, a time dependent transcript profiling was performed. A gradual upregulation of *PgGPx* was observed starting from 1h (~2 fold) to 12 h (4.5 folds) followed by a slight decrease at 24 h under 100 μM CdCl_2_ treatment (Fig 1A). Transcript abundance of *PgGPx* was maintained till 72 h with slight variation. The initial increment of *PgGPx* untill 3 h of Cd^2+^ exposure was statistically insignificant, followed by further significant enhancement at 6 h and the expression of *PgGPx* remained mostly constant till 72 h of exposure. Overall, the expression profile of *PgGPx* clearly demonstrated the imperative and prolonged role of *PgGPx* towards Cd^2+^ toxicity. The results suggest that *PgGPx* might be playing a crucial role in Cd^2+^ homeostasis.

### PgGPx possesses GST activity

Plant glutathione peroxidases belong to a versatile group of enzymes with multiple functions. It has been reported earlier that GPxs are endowed with glutathione S-transferase activity [37]. Considering the versatile nature of plant GPxs, we explicated the presence of glutathione S-transferase (GST) activity for PgGPx protein. Various enzyme kinetic parameters of PgGPx as GST activity were determined at optimum reaction conditions with a fixed concentration of CDNB in presence of a wide range of reduced glutathione (GSH). The values of *K*_m_ and *V*_max_ for GSH were found to be 11.0 mM and 526.32 nmol/mg/min with CDNB level constant (Fig 1B). Enzyme catalytic constant (*k*_cat_) and enzyme turnover (*k*_cat_/*K*_m_) were calculated from the values and were found to be 161.58 s^-1^ and 1.47 X 10^4^ M^-1^s^-1^, respectively.

### Morpho-physiological variation of *PgGPx* overexpressing transgenic rice seedlings in response to exogenous Cd^2+^ exposure

**Fig 2:**
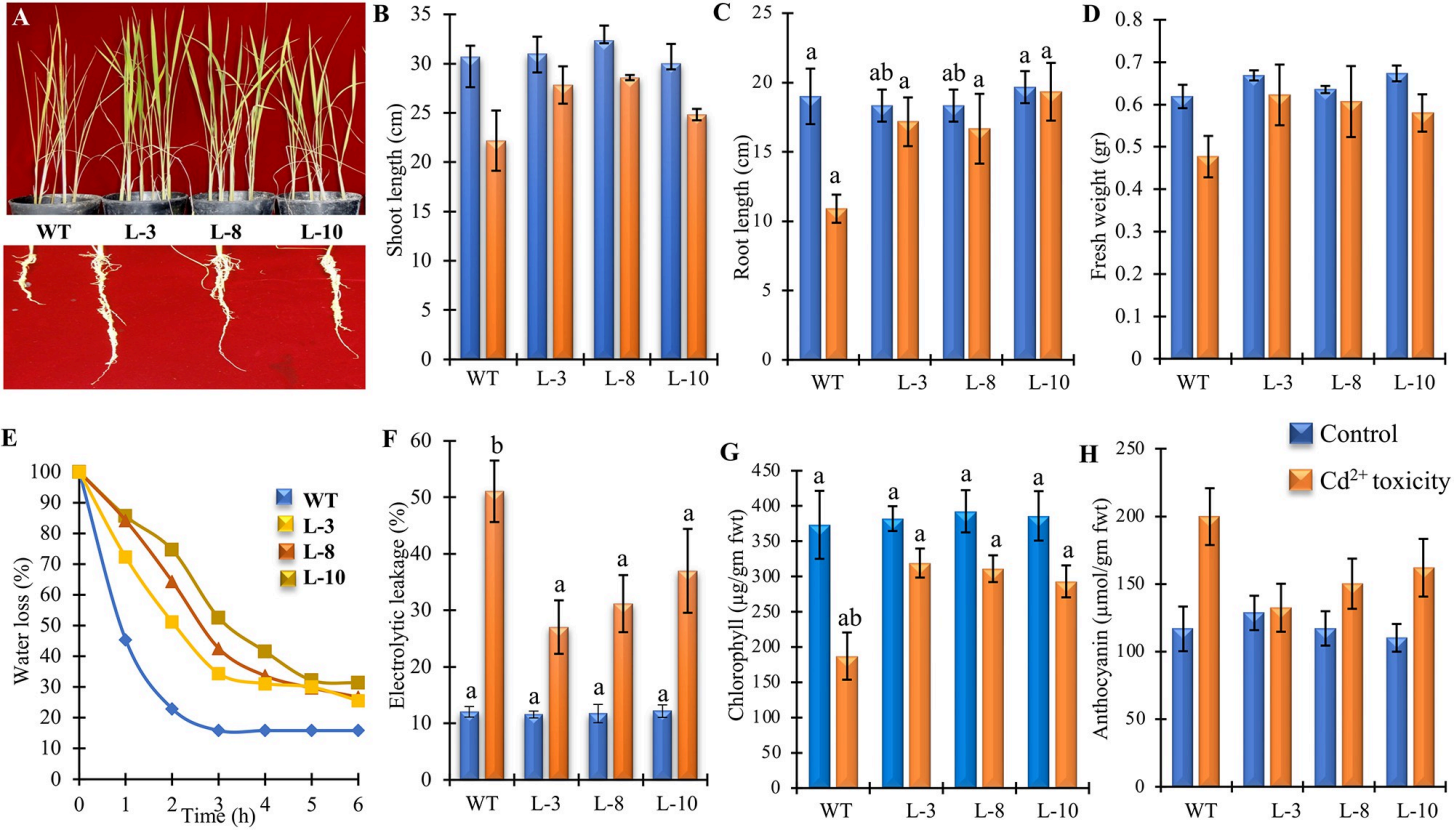
Morphological growth tolerance the ectopically expressed *PgGPx* transgenic rice plants under Cd^2+^ stress. (A) Growth of seedlings and their root of transgenic as well as WT rice plants under 100 μM CdCl_2_. Comparison of shoot length (B), root length (C), and fresh weight (D) of transgenic vis-a-vis WT plants under Cd^2+^ toxicity showed that transgenic plants had better growth potential. Analysis of water loss (E) for 6h and cellular damage through electrolyte leakage (F) in response to exogenous Cd^2+^ were measured. Total chlorophyll (G) and anthocyanin (H) contents were estimated from the transgenic and WT plants under control under stress conditions. Data represent as mean value ± standard deviation of three biological replicates (n = 3). Different letters above the bar showed significant difference between the treatments and plant genotypes (P < 0.05, ANOVA; post hoc test).

Morpho-physiological parameters such as shoot and root length, fresh biomass, and chlorophyll content are often considered as significant parameters to interpret plant growth under stress condition. Transgenic seedlings over-expressing *PgGPx* (Homologous T2 generation) were analyzed for their growth performance in response to Cd^2+^ and compared with the non-transgenic WT seedlings. Two weeks old WT and transgenic (L-3, L-8, and L-10) rice seedlings grown in vermiculite (S1 Fig) were exposed to exogenous Cd^2+^ stress. The transgenic lines showed enhanced tolerance against Cd^2+^ toxicity, while WT seedlings were unable to grow properly (Fig 2A). Moreover, the root length is an important and well documented parameter to assess plant growth inhibited due to heavy metal stress. WT plants showed a stunted root growth, while the transgenic plants were able to grow properly (Fig 2A, lower portion). In absence of Cd^2+^, no significant difference was observed in the shoot and root length of WT and transgenic plants according to ANOVA (P < 0.05) post hoc analysis (Fig 2B and 2C). Interestingly, there was no significant difference in the shoot and root length of WT and transgenic plants in presence of Cd^2+^ according to ANOVA (P < 0.05) post hoc analysis (Fig 2B and 2C). However, WT plants showed significant reduction in length in presence of Cd^2+^ as compared all three transgenic lines (Fig 2C). The level of tolerance could be directly correlated with its fresh weight. The transgenic lines revealed 5 to 14% reduction in their total fresh weight whereas, WT plants showed significant reduction of more than 20% (Fig 2D). Significant growth tolerance of transgenic plants under Cd^2+^ exposure has been supported by these data.

Relative water loss is considered as one of the most appropriate measures of plant’s water status in response to adverse condition [38]. Present study showed that *PgGPx* overexpressing plants have the capacity to maintain higher water level in comparison to WT under Cd^2+^ stress (Fig 2E). In comparison to WT, the transgenic plants were able to maintain their 50% water level until 3hr. Evaluation of the membrane integrity of cells during Cd^2+^ stress ultimately reveals their level of tolerance (Fig 2F). Conductivity measurements showed that exogenous Cd^2+^ stress affected the membrane integrity and stability in both WT and transgenic lines significantly (Fig 2F). However, WT lines showed as high as 5 folds increase in electrolyte leakage in comparison to the maximum 3 folds in transgenic lines (Fig 2F). Results suggested that transgenic plants ectopically expressing *PgGPx* gene have better membrane integrity to maintain water and electrolytes in response to Cd^2+^ toxicity.

Content of photosynthetic pigments including Chlorophyll and anthocyanin provide critical indication regarding stress perception and adaptation. Total chlorophyll content was significantly lower in WT (more than 50%) under Cd^2+^ exposure, while transgenic plants except L-10 showed a minimum reduction (Fig 2G). Further, anthocyanin content was measured from these plants. A considerable increase in anthocyanin content was observed in the WT plants, while transgenic lines except L-10 showed minimum enhancement (Fig 2H).

### *PgGPx* overexpressing lines maintain ROS homeostasis in stress

**Fig 3:**
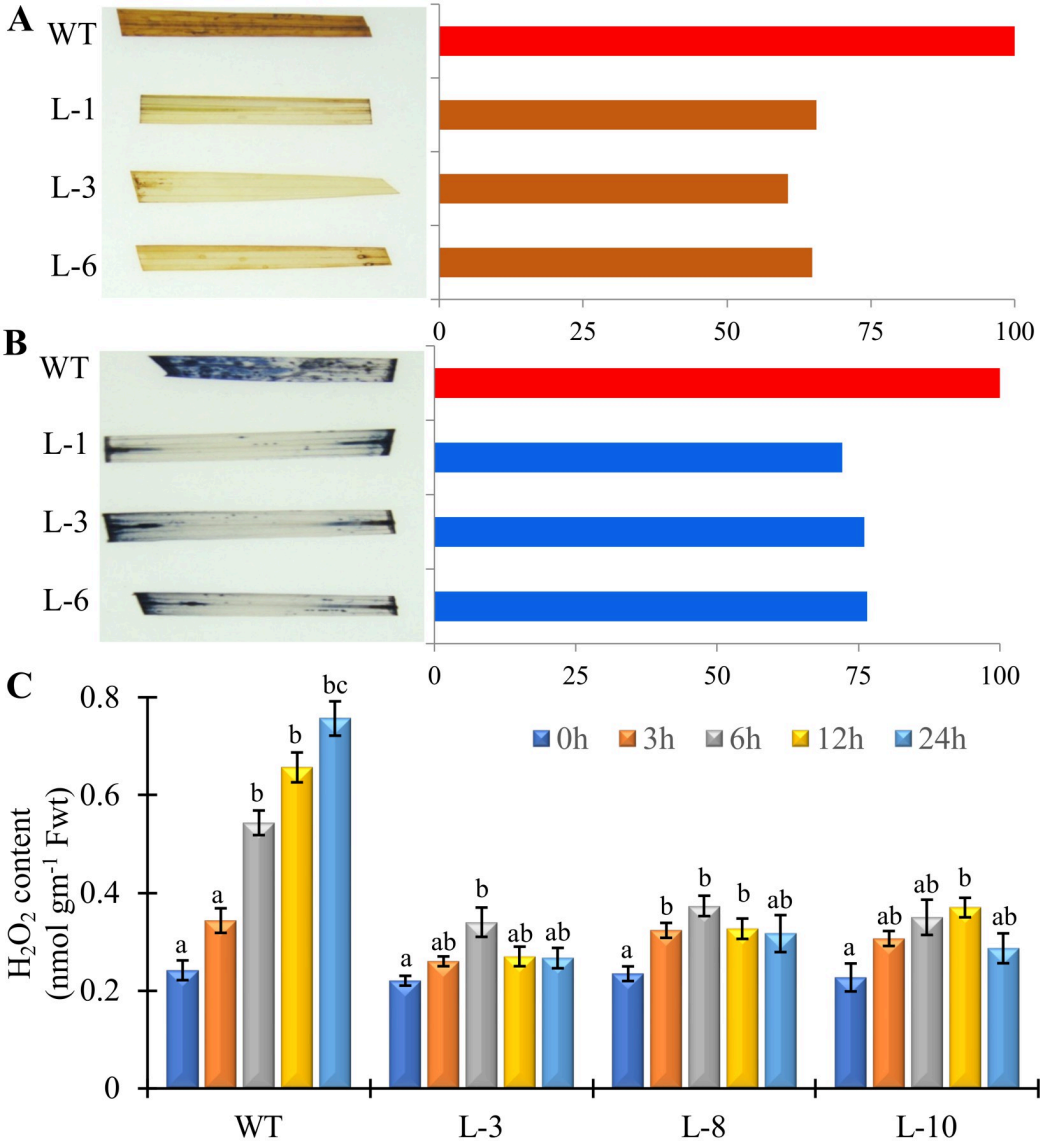
Accumulation of reactive oxygen species in the WT and *PgGPx* transgenic plants in response to exogenous Cd^2+^ stress. Accumulation of H_2_O_2_ (A) and O_2_^.-^ (B) in the transgenic and WT plants under Cd^2+^ stress was assessed by histochemical 3,3’-diaminobenzidine (DAB) and nitro blue tetrazolium (NBT) staining, respectively. (C) Total H_2_O_2_ content was measured from the transgenic and WT plants. Data represent as mean value ± standard deviation of three biological replicates (n = 3). Different letters above the bar showed significant difference between the treatments and plant genotypes (P < 0.05, ANOVA; post hoc test).

ROS homeostasis (level of H_2_O_2_ and superoxide) was analyzed in the leaves of transgenic and WT plants treated with Cd^2+^ for 48 h were measured using DAB and NBT histochemical staining (Fig 3A and 3B). It is noteworthy that less accumulation of ROS (H_2_O_2_ and superoxide O_2_^.-^) occurs in all three tested transgenic lines in contrast to WT (Fig 3A and 3B). Visual observation of ROS staining (Fig 3A and 3B, left panel) was quantitatively verified by densitometric scanning of the image and represented as bar diagram (Fig 3A and 3B, right panel). Both the visual observation and densitometric data confirmed the higher accumulation of ROS in the WT plants compared to *PgGPx* overexpressing transgenic lines under Cd^2+^ toxicity. Furthermore, the level of total H_2_O_2_ was measured from the WT and transgenic plants under Cd^2+^ for 24 hr (Fig 3C). WT plants showed a gradual accumulation H_2_O_2_ over time and reached to the peak of almost four times higher than the 0 h value, whereas the transgenic lines showed only 1.5 to 2 folds increment (Fig 3C).

### Analyses of ROS scavenging enzymes in *PgGPx* overexpressing lines under Cd^2+^ toxicity

**Fig 4:**
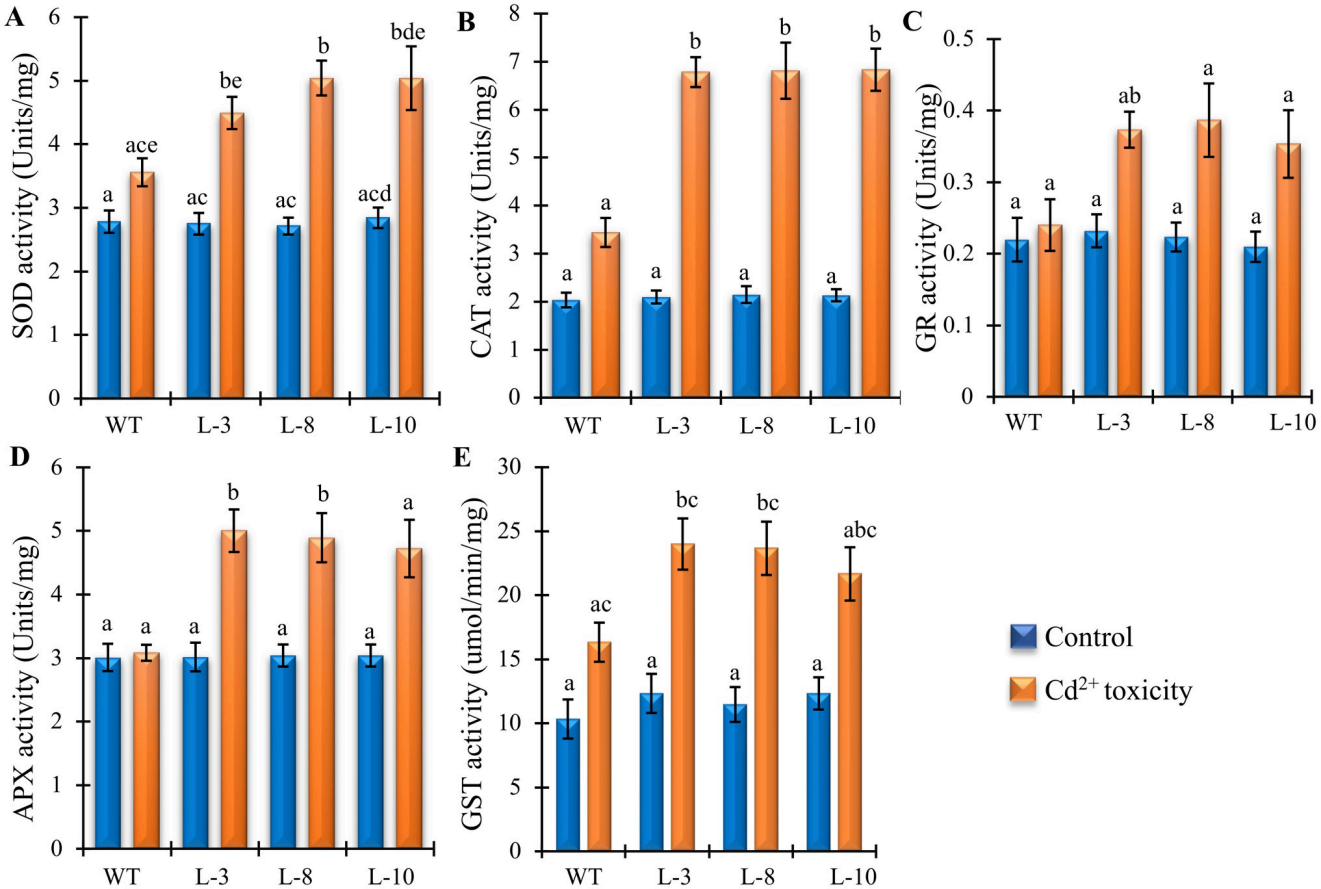
Effects of Cd^2+^ stress on the activity of antioxidant enzymes. Activities different antioxidant enzymes including (A) Superoxide dismutase (SOD), (B) Catalase (CAT), (C) Glutathione Reductase (GR), (D) Ascorbate peroxidase (APX) and (E) Glutathione S-transferase (GST) were measured from WT and transgenic plants under control condition and exposed to 100 μM CdCl_2_. Data represent as mean value ± standard deviation of three biological replicates (n = 3). Different letters above the bar showed significant difference between the treatments and plant genotypes (P < 0.05, ANOVA; post hoc test).

Antioxidant enzymes such as superoxide dismutase (SOD), ascorbate peroxidase (APX), catalase (CAT), glutathione S-transferase (GST), peroxidases and glutathione reductase (GR) are closely related to the accumulation of ROS. The key metabolic steps during ROS generation are controlled by these enzyme families in eukaryotic systems. Cd^2+^ stress-induced changes in the antioxidant enzyme activities have been analyzed in the transgenic and WT plants. When the heavy metal concentration inside the cell crosses the threshold level, the product of ROS is induced. In control condition, the specific activities of antioxidant enzymes i.e. SOD, APX, CAT and GR were found to be similar in both transgenic and WT plants according to ANOVA post hoc analysis that indicated a similar state of antioxidative cellular environment in both types of plants (Fig 4A–4D). Interestingly, after exposure to exogenous Cd^2+^, the levels of SOD, APX, CAT and GR activities increased significantly in the most of transgenic lines in comparison to WT (Fig 4A–4D). As Cd^2+^ stress modulated the overall antioxidant enzyme activities in transgenic lines, *PgGPx* overexpressing transgenic plants amended or suppressed the Cd^2+^ metal induced oxidative stress. In case of GR (Fig 4C) and APX (Fig 4D), WT plants didn’t show any significant enhancement in the enzyme activity after Cd^2+^ toxicity, while the transgenic plants showed significant accumulation of antioxidant enzymes.

Here, we have reported that PgGPx possessed GST activity along with conventional GPx activity (Fig 1B). The heterologous expression of *PgGPx* in rice, thus direct enhanced the level of GST activity under control condition (Fig 4E). Interestingly, both WT and *PgGPx* overexpressing plants showed significant enhancement of GST activity in response to Cd^2+^ stress. But the transgenic lines showed significantly high levels of GST activity which ultimately resulted in the manipulation of heavy metal homeostasis. It is clearly observed that transgenic plants had not only increased GPx/GST activity but all antioxidant enzyme activities under heavy metal exposure compared to WT plants.

### Effect of heavy metal toxicity on the photosynthetic machinery and overall yield of plants

**Fig 5:**
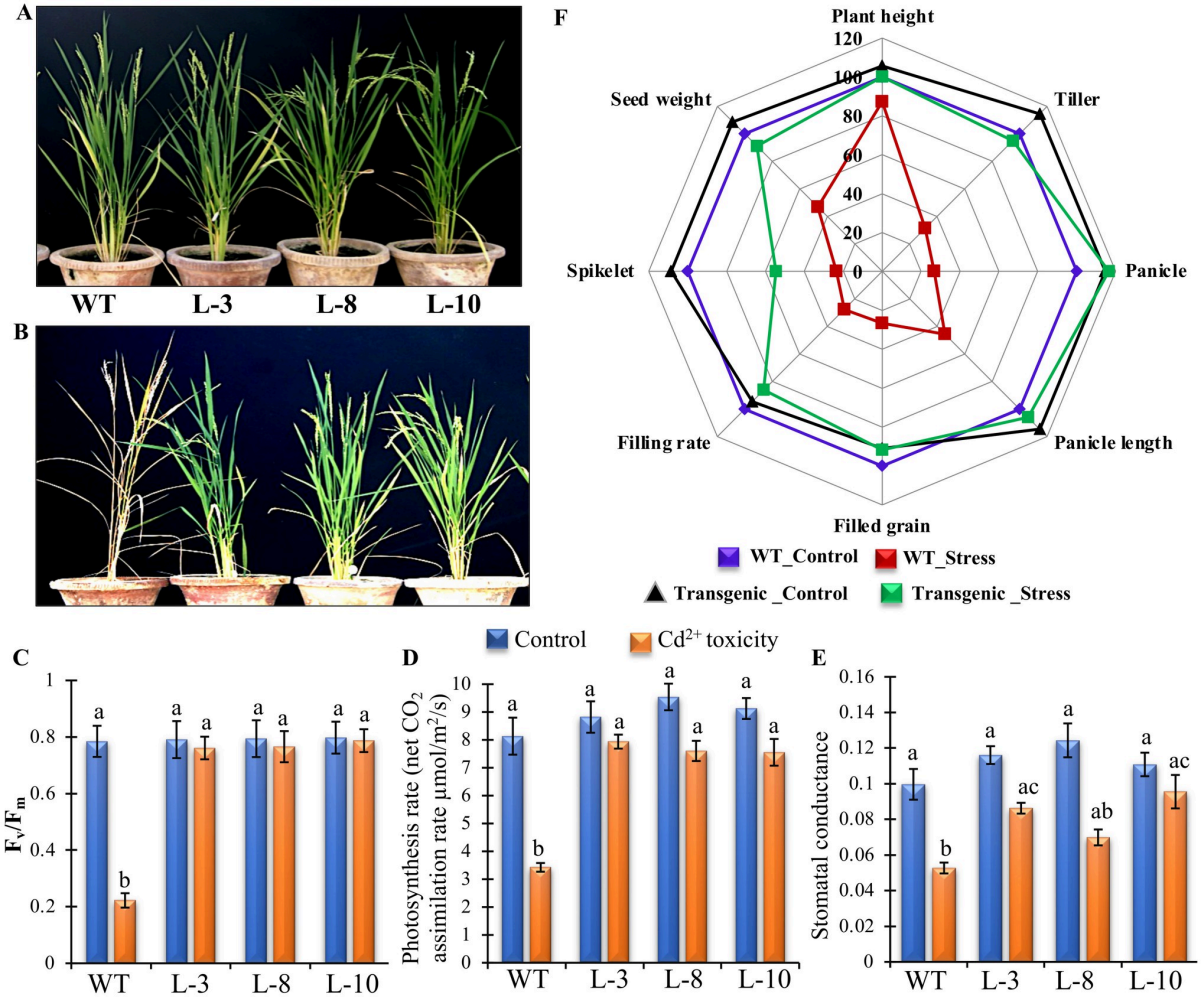
Photosynthetic and yield-related outcomes under Cd^2+^ toxicity. Mature WT as well as *PgGPx*-transgenic plants grown in normal condition (A) or in presence 100 μM CdCl_2_ (B). Different photosynthetic parameters including photosystem II efficiency through Fv/Fm (C), rate of photosynthesis (D), and stomatal conductance (E) were measured for three independent transgenic lines and WT plants under control and Cd^2+^ stress conditions. Data represent as mean value ± standard deviation of three biological replicates (n = 3). Different letters above the bar showed significant difference between the treatments and plant genotypes (P < 0.05, ANOVA; post hoc test). (F) Spider plots of different agronomic traits including plant height, tiller number, panicle number, panicle length, number of filled grain, grain filling rate, number of spikelets, and seed weight for *PgGPx*-overexpressing rice transgenic plants (average of three transgenic lines) were compared to the WT plants grown under normal and stress conditions. WT grown under control condition were considered as 100%.

Two-months-old rice plants were treated with exogenous Cd^2+^ until maturity along with control to evaluate the effect of heavy metal toxicity on the total yield. In control condition, both WT and transgenic plants grew normally with similar pattern for seed sets and maturation (Fig 5A). However, WT plants were not able to survive under heavy metal exposure; whereas the transgenic plants were able to grow and form seed in adverse condition (Fig 5B). Photosynthetic capacity of plants is largely responsible on the overall growth status of plants. Physiological effect of heavy metal toxicity is directly proportional to the percentage loss of photosynthetic pigments. *PgGPx* over-expressing transgenic lines retained higher percentage of chlorophyll content (almost 90%) than corresponding WT with less than 50% chlorophyll content (Fig 2G). Apart from the pigmentation, other assorted photosynthetic parameters including efficiency of photosystem II (PSII) through Fv/Fm, rate of photosynthesis, and stomatal conductance were measured and compared (Fig 5C–5E). All the transgenic and WT plants showed similar Fv/Fm values under control condition indicating no alteration of PSII efficiency in the transgenic lines (Fig 5C). A sharp decrease in the Fv/Fm ratio was observed in case of WT plants in response to Cd^2+^, while the transgenic lines maintained significantly higher Fv/Fm ratio during stress (Fig 5C). Similarly, net photosynthetic rate is in harmony with the pigments count in context to WT and transgenic lines (except L-8) under control condition (Fig 5D). Moreover, transgenic plants maintained the photosynthetic efficiency significant better than the WT lines under stress condition (Fig 5D). In addition, photosynthetic rate is affected by the rate of stomatal conductance because it plays a crucial role in maintaining the equilibrium between transpiration and CO_2_ absorption rates. It was observed that stomatal conductance levels lower significantly in WT plants compared to *PgGPx* overexpressing transgenic lines (Fig 5E). Altogether, *PgGPx* transgenic lines exhibited similar photosynthetic rate under control condition, compared to the WT plants with few exceptions. But transgenic plants were able to maintain all the photosynthetic related parameters with minimum fluctuation compared to the drastic reduction of WT plants under Cd^2+^ toxicity. This might lead towards better yield outcome of transgenic lines as compared to WT plants under stress conditions (Fig 5F).

Relative yield potential (different parameters including plant height, number of tillers, panicle number and length, spikelet number, filled grain rate, number of filled grains and average seed weight) of the transgenic lines along with WT under control and Cd^2+^ exposure was assessed, and compared (Fig 5F). Comparative results revealed that the transgenic plants produced either similar or better yield under control condition compared to WT. WT plants exhibited only 25–30% filled grain under heavy metal stress compared to its control condition, whereas the transgenic plants had no grain number penalty (Fig 5F). A significant decrease in the number of tilers, spikelet, panicle, and grain was observed in WT plants under stress conditions unlike the transgenic plants, those were able to maintain all the estimated parameters including grain filling rate and filled grain with minimum reduction. These transgenic plants were also able to produce similar amount of yield like WT plants under control condition. Therefore, overexpression of *PgGPx* gene might help plant to overcome the Cd^2+^ stress induced cellular and physiological damages with minimum yield penalty.

## Discussion

Accumulation of phytotoxic metals such as zinc, copper, iron and Cd^2+^ due to the industrial and agricultural malpractices, may reduce plant growth and productivity. These widespread pollutants damage plants via two different modes: a) direct inhibition of plant growth and biosynthetic pathways, and b) involvement in free radical production [39]. In a previous study, we have found that PgGPx is a Cd^2+^-dependent, functional peroxiredoxin [25]. Since, accumulation of excess Cd^2+^ might be toxic to plant as well as human if remain in the edible parts of plants, presence of a Cd^2+^-dependent enzyme (PgGPx) might be involved in maintaining the homeostasis of Cd^2+^ ions. A continuous enhancement of *PgGPx* transcript was observed in response to exogenous Cd^2+^ treatment until 6 h, followed by a steady level till 72 h of observed period (Fig 1A). Interestingly, we have observed that PgGPx possessed GST activity along with its conventional GPx activity as a dual function (Fig 1B). All these findings prompted us to analyze the ectopically expressing *PgGPx* transgenic rice plants in responses to toxic metal Cd^2+^.

Excessive Cd^2+^ causes oxidative stress by generating ROS (superoxide radicals, O_2_^.-^ and hydrogen peroxide, H_2_O_2_), therefore causing damage to DNA, modifying protein side chains, destroying phospholipids that ultimately leads to the reduction of plant growth and development [20]. Altered physiological phenomena such as reduce root length and total fresh weight in response to toxic levels of Cd^2+^ have been reported previously [40]. Classical symptoms of Cd^2+^ stress are short roots, leaf chlorosis, early senescence with reduced biomass [41–43]. Transgenic rice plants over-expressing *PgGPx* provided considerable growth tolerance under Cd^2+^ stress (Fig 2) indicating towards the possible role of *PgGPx* in combating Cd^2+^ toxicity. Chlorophyll being the major biological pigment in plants depicts their growth, nutritional status, and crop productivity. Weakening of pigment biosynthetic pathway is closely associated with chlorosis and growth retardation in response to Cd^2+^ exposure and it is well established that Cd^2+^ induces depletion of chlorophyll content in a variety of plants [44, 45]. In contrast, a significantly higher total chlorophyll was observed in our study on *PgGPx* overexpressing transgenic rice plants under Cd^2+^ stress as compared to the WT plants (Fig 2G). This might be due to the possibility of PgGPx mediated up-regulation of several enzymatic antioxidants (Fig 4), thereby maintaining the ROS homeostasis for normal growth in response to Cd^2+^ stress. Several reports indicated that Cd^2+^ has role in several stress tolerance signal transduction pathways and eventually modulate the expression of a large number of stress-responsive genes to provide stress tolerance [1]. Several biochemical pathways: (i) induction of oxidative stress; (ii) interference with signalling pathways; and (iii) interference with DNA repair are the integral part of the molecular mechanisms of cellular Cd^2+^ toxicity [46].

During respiration and photosynthesis plants generate ROS as a by-product of normal cellular metabolism during electron transport. Under heavy metal stress, ROS produced as the first line of defense in plants. But excess level of ROS causes damage to proteins, lipids, carbohydrates, DNA and eventually leads to cell death [47, 48]. Moreover, chlorophyll degradation and inhibition or stimulation of the activity of several antioxidant enzymes escalates under Cd^2+^ exposure [49, 50]. Activity of antioxidative enzymes like APX, SOD, CAT, GR, and GST were enhanced in *PgGPx* transgenic lines as compared to the non-transgenic WT plants in response to Cd^2+^ stress (Fig 4). These genes are the key players of Ascorbate-glutathione pathway and during stress plays a crucial role in combating oxidative stress. Besides, it was observed that ectopically *PgGPx* expressive transgenic rice plants showed minimum reduction in most of the photosynthetic machinery components including Fv/Fm, photosynthesis rate, and stomatal conductance (Fig 5C–5E) and thus, were able to maintain most of the yield parameters within a considerable range under Cd^2+^ stress (Fig 5F).

In the present study, *PgGPx* overexpressed transgenic rice lines showed significant tolerance against Cd^2+^ stress. The possible explanation could be the internal mechanism for Cd^2+^ transportation and sequestration along with the improvement of photosynthetic machinery and antioxidant enzyme cascades play a significant role for this phenomenon. Previously PgGPx has been reported to exhibit peroxidase activity in both glutathione and thioredoxin dependent manner [25]. In GSH dependent cycle, PgGPx helps to recycle reduced GSH, which in turn promotes synthesis of phytochelatins. Phytochelatins are key troupe in metal sequestration to vacuole or removal of metal ions from the cell. This observation is concomitant with heavy tolerance by a GPx-GST dual functional enzyme. Although overexpression of *PgGPx* provides Cd^2+^ tolerance, further detailed studies are required to explain the entire complex scenario.

## Conclusion

Taken together, overexpression of *PgGPx* in rice plants provides significant tolerance against Cd^2+^ toxicity via restoring and maintaining adequate cellular ion homeostasis as well as modulation of antioxidant enzyme activity to reduce the oxidative stress. The transgenic rice plants displayed enhanced growth phenotypes such as higher biomass, root growth and photosynthetic pigments with no yield penalty in comparison to WT. Hence, the present data suggests that *PgGPx* overexpressing transgenic rice plants are better equipped to survive in heavy metal contaminated environments. All these results reveal to be a potential candidate gene for enhancing agricultural productivity of important crops particularly rice.

## Supporting information

S1 Text Complete sequence of *PgGPx* gene. (PDF)

S1 Table Primers used in the study for real time PCR. (PDF)

S1 Fig Seedlings of WT and *PgGPx* overexpressing rice under control condition. Seedlings from WT and three transgenic lines were germinated and grown on vermiculite under control condition.
(PDF)

S1 File Raw real-time data and calculation. (XLSX)
