# Supplementary material for: Evaluation of Cd2+ stress tolerance in transgenic rice overexpressing PgGPx gene that maintains cellular ion and reactive oxygen species homeostasis
Source: PLoS One. 2022 Sep 6;17(9):e0273974. doi: 10.1371/journal.pone.0273974 (PMC9447883; doi:10.1371/journal.pone.0273974)
Supplement: S1 Text — (PDF) [file pone.0273974.s001.pdf]

S1 Text. Complete sequence of *PgGPx* gene

ATGGCTGCTGCTTCGTCCGCCGCCTCCGTCCACGATTTCACCGTCAAGGATGCAAGCGGGAA
GGATGTTGACCTCAGCACCTACAAGGGCAAGGTTCTCCTTATTGTCAACGTCGCATCCCAGT
GTGGATTAACTAACTCCAACTACACCGAGCTGGCCCAGCTCTATGAGGAGTACAAGGACCAA
GGTTTTGAGATCCTGGCTTTTCCTTGCAACCAGTTTGGAGGGCAGGAGCCTGGCACAAACGA
GGAGATTGTGCAGTTTGCTTGCACACGCTTCAAGGCTGAGTACCCCATCTTCGACAAGGTTA
ATGTCAACGGTAACGATGCAGCGCCCATCTACAAGTTCCTGAAGTCTAGCAAGGGTGGCCTC
TTCGGTGACAGCATCAAGTGGAACTTCTCCAAGTTCTTGGTTGACAAGGAGGGGCGTGTTGT
GGATCGCTATGCCCCGACCACTTCCCCCCTGAGCATAGAGAAGGATATCAAGAAACTACTCG
GAAGTTCTTAA
