# Supplementary material for: Evaluation of Cd2+ stress tolerance in transgenic rice overexpressing PgGPx gene that maintains cellular ion and reactive oxygen species homeostasis
Source: PLoS One. 2022 Sep 6;17(9):e0273974. doi: 10.1371/journal.pone.0273974 (PMC9447883; doi:10.1371/journal.pone.0273974)
Supplement: S1 Table — (PDF) [file pone.0273974.s002.pdf]

S1 Table. Primers used in the study for real time PCR

**Sl no**	**Primer name**	**Sequence (5'- 3')**
**1.**	*PgGPx*-Forward	ACCTTGTAAACTGGAGCCGC
**2.**	*PgGPx*-Reverse	CATTGCATTGAACCTGATGC
**3.**	*Tubulin*-Forward	GTGCTCTGAATGTGGATGTGAATG
**4.**	*Tubulin*- Reverse	ACCAACCTCCTCATAGTCCTTCTC
