# Supplementary material for: Evaluation of Cd2+ stress tolerance in transgenic rice overexpressing PgGPx gene that maintains cellular ion and reactive oxygen species homeostasis
Source: PLoS One. 2022 Sep 6;17(9):e0273974. doi: 10.1371/journal.pone.0273974 (PMC9447883; doi:10.1371/journal.pone.0273974)
Supplement: S1 Fig — Seedlings from WT and three transgenic lines were germinated and grown on vermiculite under control condition. (PDF) [file pone.0273974.s003.pdf]

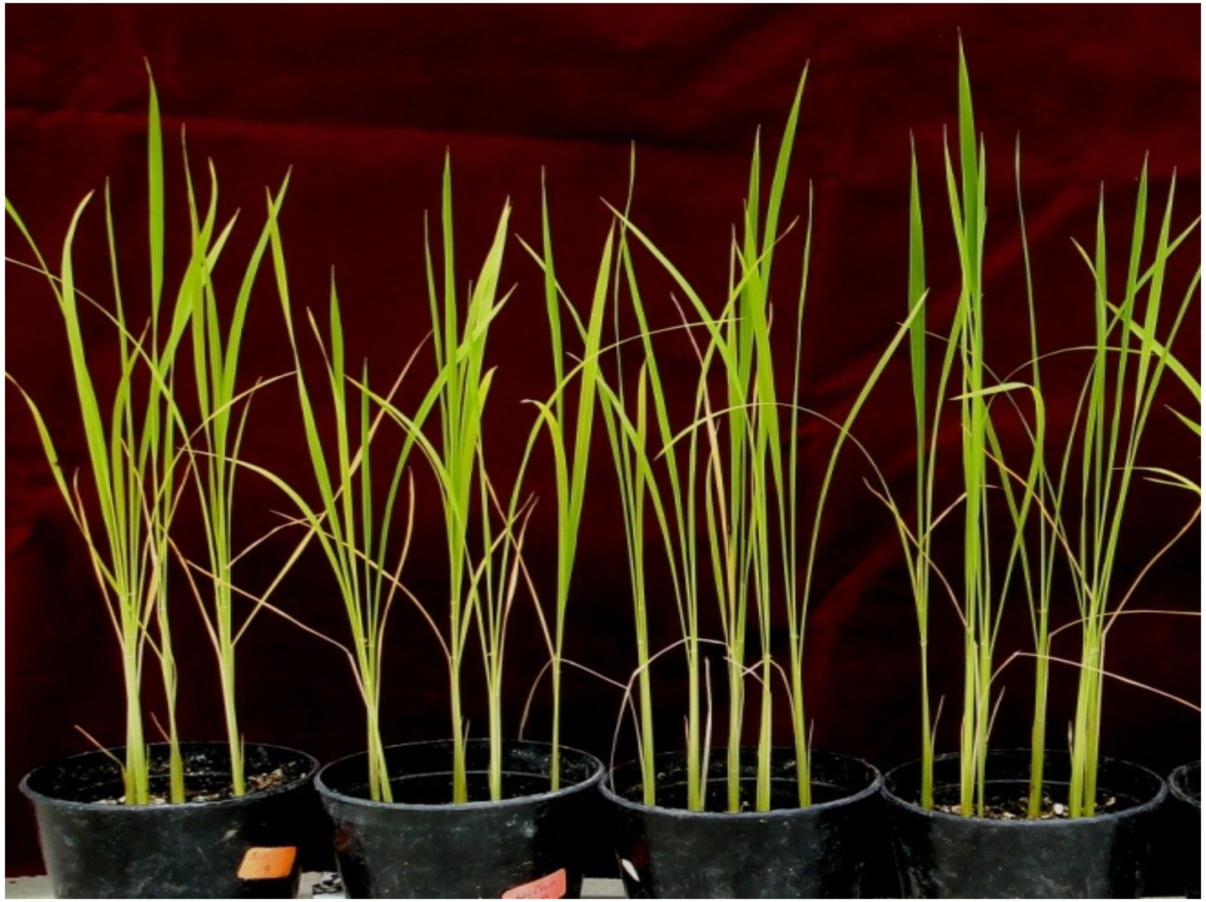

WT L-3 L-8 L-10

S1 Fig. Seedlings of WT and *PgGPx* overexpressing rice under control condition. Seedlings from WT and three transgenic lines were germinated and grown on vermiculite under control condition.
